# Clinical assessment of peripheral perfusion to predict postoperative complications after major abdominal surgery early: a prospective observational study in adults

**DOI:** 10.1186/cc13905

**Published:** 2014-06-03

**Authors:** Michel E van Genderen, Jorden Paauwe, Jeroen de Jonge, Ralf JP van der Valk, Alexandre Lima, Jan Bakker, Jasper van Bommel

**Affiliations:** 1 Department of Intensive Care, Erasmus MC, ‘s Gravendijkwal 230, 3015 CE Rotterdam, the Netherlands; 2 Department of Surgery, Erasmus MC, ‘s Gravendijkwal 230, 3015 CE Rotterdam, the Netherlands; 3 Department of Epidemiology, Erasmus MC – Sophia’s Children Hospital, Wytemaweg 80, 3015 CN Rotterdam, the Netherlands; 4 Department of Paediatrics, Erasmus MC, Wytemaweg 80, 3015 CN Rotterdam, the Netherlands

## Abstract

**Introduction:**

Altered peripheral perfusion is strongly associated with poor outcome in critically ill patients. We wanted to determine whether repeated assessments of peripheral perfusion during the days following surgery could help to early identify patients that are more likely to develop postoperative complications.

**Methods:**

Haemodynamic measurements and peripheral perfusion parameters were collected one day prior to surgery, directly after surgery (D0) and on the first (D1), second (D2) and third (D3) postoperative days. Peripheral perfusion assessment consisted of capillary refill time (CRT), peripheral perfusion index (PPI) and forearm-to-fingertip skin temperature gradient (T_skin-diff_). Generalized linear mixed models were used to predict severe complications within ten days after surgery based on Clavien-Dindo classification.

**Results:**

We prospectively followed 137 consecutive patients, from among whom 111 were included in the analysis. Severe complications were observed in 19 patients (17.0%). Postoperatively, peripheral perfusion parameters were significantly altered in patients who subsequently developed severe complications compared to those who did not, and these parameters persisted over time. CRT was altered at D0, and PPI and T_skin-diff_ were altered on D1 and D2, respectively. Among the different peripheral perfusion parameters, the diagnostic accuracy in predicting severe postoperative complications was highest for CRT on D2 (area under the receiver operating characteristic curve = 0.91 (95% confidence interval (CI) = 0.83 to 0.92)) with a sensitivity of 0.79 (95% CI = 0.54 to 0.94) and a specificity of 0.93 (95% CI = 0.86 to 0.97). Generalized mixed-model analysis demonstrated that abnormal peripheral perfusion on D2 and D3 was an independent predictor of severe postoperative complications (D2 odds ratio (OR) = 8.4, 95% CI = 2.7 to 25.9; D2 OR = 6.4, 95% CI = 2.1 to 19.6).

**Conclusions:**

In a group of patients assessed following major abdominal surgery, peripheral perfusion alterations were associated with the development of severe complications independently of systemic haemodynamics. Further research is needed to confirm these findings and to explore in more detail the effects of peripheral perfusion–targeted resuscitation following major abdominal surgery.

## Introduction

Despite reductions in postoperative mortality, the occurrence of severe complications remains high [1]. The development of postoperative complications affects the prognosis of surgical patients and substantially increases the utilization of resources and the cost of care [1-3]. Early recognition of patients more likely to develop postoperative complications is therefore of prime importance. Because postoperative complications better predict short- and long-term mortality than preoperative and intraoperative factors [3-5], recent research has been focused on identifying preoperative factors that predispose patients to postoperative complications. Several scoring systems, such as the Acute Physiology and Chronic Health Evaluation II (APACHE II) score, the American Society of Anesthesiologists (ASA) score and the Portsmouth Physiological and Operative Severity Score for the enUmeration of Mortality and Morbidity (P-POSSUM) score can be applied in a general surgery population. These scores are based on preoperative and perioperative variables specific to different types of surgery. Despite the great number of identified predictors, these scores do not take into consideration the individual patient’s postoperative situation, are difficult to calculate [6], cannot be calculated over time and are still doubted for their specific predictive value for assessing the individual high-risk surgery patient [7]. Therefore, in clinical practice, a simple, easy-to-use approach is needed to recognize patients at risk for severe complications and to ensure timely initiation of interventions to improve outcomes.

There is increasing evidence that altered tissue perfusion in high-risk surgical patients could be helpful for the detection of those at risk for complications [8,9] and optimally improve outcomes [10]. In this regard, the success of early, goal-directed haemodynamic therapy has demonstrated the importance of maintaining and improving tissue oxygenation and has shown that early detection and correction of altered tissue perfusion reduce postoperative complications [2,11]. Accordingly, their importance is also the basis for stressing the need to monitor postoperative early warning signals for occult tissue hypoperfusion [2,12]. Likewise, lactate level, a potential marker of occult hypoperfusion, is used as a resuscitation target, although its relationship with regional circulation is still not clear [13,14]. Therefore, early recognition of regional tissue perfusion abnormalities remains important to avoid further organ damage and improve outcomes following major surgery. Postoperative monitoring is still based on conventional haemodynamic variables, which are known to be insensitive to determination of the presence of regional tissue hypoperfusion.

Recently, we and others have shown that assessment of perfusion of the peripheral circulation enables the identification of patients who will have unfavourable outcomes. In critically ill patients [15], after out-of-hospital cardiac arrest [16] and during septic shock [17], impaired peripheral perfusion has been shown to be associated with organ failure and increased mortality. By use of the capillary refill time (CRT), the peripheral perfusion index (PPI) (Masimo SET Radical-7 pulse oximeter on a rainbow and SatShare platform; Masimo UK, Basingstoke, UK) and the forearm-to-fingertip body temperature gradient (T_skin-diff_), peripheral perfusion can easily and noninvasively be evaluated at the bedside and may thus be a more simple and generally useful tool for identifying patients at risk for postoperative complications [18]. In this context, we were interested in determining whether repeated assessment of the peripheral circulation in the days following surgery could help in the early identification of patients who are more likely to develop postoperative complications. We hypothesized that a disturbance of the peripheral perfusion might be present more frequently in patients who develop severe complications after major abdominal surgery.

## Material and methods

### Study population

We conducted this single-centre, prospective observational study between September 2011 and June 2012. We included consecutive patients scheduled to undergo major abdominal surgery. All procedures were performed by senior surgeons and were defined as any intervention in which extensive resection was performed, a body cavity was entered, organs were removed or normal anatomy was significantly altered. Operations included colorectal, gastric, hepatic, pancreatic and oesophageal surgery for benign and malignant disease. Patients were suitable for inclusion if they met the following criteria: (1) age ≥18 years, (2) ASA Physical Status between 1 and 4 and (3) expected duration of surgery ≥120 minutes. Patients were excluded if they met the following criteria: (1) known neurologic or peripheral arterial occlusive disease, (2) refusal of consent, (3) pregnancy, (4) emergency surgery or (5) minor abdominal surgery. Medical ethical approval was provided by the Human Research Ethics Committee of the Erasmus University Medical Centre (Erasmus MC), Rotterdam, the Netherlands. Written informed consent was obtained from all patients at least 1 day prior to surgery.

### Data collection

Haemodynamic variables, metabolic state and peripheral perfusion parameters were collected 1 day before surgery (BL), directly after surgery (D0) and on the first (D1), second (D2) and third (D3) postoperative days. Before surgery, basic demographic characteristics and routine biological, standard haemodynamic and peripheral perfusion parameters were recorded. Standard haemodynamic monitoring included continuous recording of electrocardiographic data, heart rate (HR), mean arterial pressure (MAP), body temperature and pulse oximetry. Concurrently at each time point, arterial blood samples were taken for blood gas analysis, arterial haemoglobin and lactate concentration (Radiometer Copenhagen ABL700 blood gas analyser; Radiometer Medical, Copenhagen, Denmark). Additionally, surgery duration, operative blood loss, vasopressor therapy (any dose of norepinephrine), ICU and hospital length of stay, length of ventilator support and 30-day mortality were recorded.

We assessed all data needed to calculate the APACHE II score [19], Sequential Organ Failure Assessment (SOFA) score [20] and P-POSSUM score [21]. The total SOFA score was calculated by summing the scores for each of the components (that is, cardiac, renal, respiratory, coagulation, and liver). We did not record the data for second surgeries in patients who underwent reoperation during the same hospitalization.

### Definition of complications

Complications were defined as the presence of complications within the first 10 days after surgery. We also scored in-hospital 30-day postoperative complications. Postoperative complications were graded according to the Clavien-Dindo classification system [22,23]. In short, grades I and II complications are defined as any deviation from the normal postoperative course, without the need for surgical, endoscopic or radiological interventions (grade I) but with the need for pharmacological treatment (grade II). Grade III complications required a surgical, endoscopic or radiological intervention during local (grade IIIa) or general anaesthesia (grade IIIb). Grade IV complications include those requiring ICU care because of single organ failure (grade IVa) or multiorgan failure (grade IVb). Postoperative overnight monitoring in the ICU was routinely performed in patients after oesophagectomy with gastric tube reconstruction and after liver transplantation, and therefore was not scored as a complication *per se*, unless a complication, as described in the Clavien-Dindo classification scheme, occurred and prolonged expected ICU stay, or in cases where continuous ICU admission was necessary for treatment. Additionally, we recorded 30-day survival to evaluate grade V complications (that is, death during the postoperative period). Grades III and IV complications and death (grade V) are classified as severe complications [24].

In addition, we defined infectious complications as one of the following infections: pneumonia (infiltrate noted on chest radiograph or positive sputum culture), sepsis and related syndromes according to the international sepsis consensus guidelines [25], urinary tract infection (white blood cells in urine) and wound infection. Leakage was defined as anastomotic or chyle leakage. Pleural effusion was diagnosed by chest radiography and/or computed tomography.

### Peripheral perfusion assessment

Peripheral perfusion was evaluated using the CRT, the PPI and the forearm-to-fingertip (T_skin-diff_) body temperature gradient. These methods are more extensively described elsewhere [18]. In short, CRT is defined as the time required for a distal capillary bed (that is, the nail bed) to regain its colour after pressure has been applied to cause blanching. The time to return of normal colour was measured with a per-second analog hospital clock, which was present in every hospital room. A delayed return to normal colour (>5 seconds) is regarded as impaired peripheral perfusion and has been related to tissue hypoperfusion and an increased likelihood of worsening organ failure [15]. Additionally, to investigate the reliability of CRT, which is a subjective assessment, in terms of variability between different health-care workers, two examiners evaluated CRT in each patient—a trained researcher and a random nurse at the ward at the concurrent time point.

The PPI, which we measured using the Masimo SET Radical-7 pulse oximeter on a rainbow and SatShare platform, provides a noninvasive indicator of the peripheral vasomotor tone and peripheral perfusion and is derived from the photoelectric plethysmographic signal of the pulse oximeter, which is placed on the finger. A threshold value of 1.4 represents a very sensitive cutoff point for determining abnormal peripheral perfusion associated with vasoconstriction [26].

T_skin-diff_ values was obtained from two skin probes attached to the index finger and on the radial side of the forearm, midway between the elbow and the wrist. This temperature gradient can reflect changes in cutaneous blood flow better than the absolute skin temperature itself and is related to blood flow. T_skin-diff_ increases during vasoconstriction. A threshold value of 2°C has been shown to reflect intermediate vasoconstriction, and a threshold >4°C reflects severe vasoconstriction, in critically ill patients [15].

### Statistical analysis

Data are presented as mean ± SE, unless otherwise specified. We used a Kolmogorov–Smirnov test to test for normality (*P* > 0.05). Differences between group means were tested by Student’s *t*-test or Mann–Whitney *U* test. To analyse changes in the different systemic haemodynamic and biological variables over time and between groups, we used linear mixed-model analysis. We retrospectively divided the groups into group A (nonsevere) and group B (severe) complications. Briefly, patients without complications and grades I and II complications were combined as group A, and grades III, IV and V complications were labelled group B. Group B complications (grades III to V) are known as severe complications because of the necessity of surgical intervention (grade III) or due to their severity (that is, organ failure (grade IV or death (grade V)) and are therefore defined as the primary outcome [6,24]. Because peripheral perfusion parameters are quantitative results, cutoff values were necessary to determine outcome associations. We therefore used predefined cutoffs for abnormal peripheral perfusion (cutoffs: CRT >4.5 seconds, PPI <1.4 and T_skin-diff_ >2°C) to evaluate the relationship with outcome [15,18]. We then constructed receiver operating characteristic (ROC) curves (plotted as continuous variables). To calculate sensitivity, specificity, positive likelihood ratio and negative likelihood ratio, we used the predefined cutoffs for abnormal perfusion. We also performed a generalized mixed-model analysis to calculate the predictive value, which was estimated using odds ratios (ORs) with 95% confidence intervals (CIs), for each postoperative day. This multivariate model was confirmed by using forward stepwise selection. We selected the variables based on differences between groups and on previous reports of prognostic factors for peripheral perfusion [15,27]. We therefore corrected for MAP, HR, vasopressor therapy, C-reactive protein (CRP) and haemoglobin. We used severe complications as our dichotomous outcome variable and any combination of abnormal peripheral perfusion parameters as our dichotomous predictor variable. Subsequently, we performed binary logistic regression to further explore the predictive value of the more traditional predictive scores (APACHE II, ASA, P-POSSUM and SOFA scores). The Bonferroni correction was applied to correct for multiple testing, assuming an α of 0.05 for the three independent tests (CRT, PPI and T_skin-diff_). Therefore, *P*-values <0.017 were considered statistically significant.

Interobserver variability for subjective assessment of peripheral perfusion based on CRT was assessed by calculating Cohen’s κ (clinical cutoff >5 seconds). A κ-value ≥0.7 was regarded as adequate. Most analyses were conducted using SPSS version 20.0 software (SPSS, Chicago, IL, USA). The ROC analyses were performed in SigmaPlot 11.0 software (Systat Software, San Jose, CA, USA).

## Results

**Figure 1:**
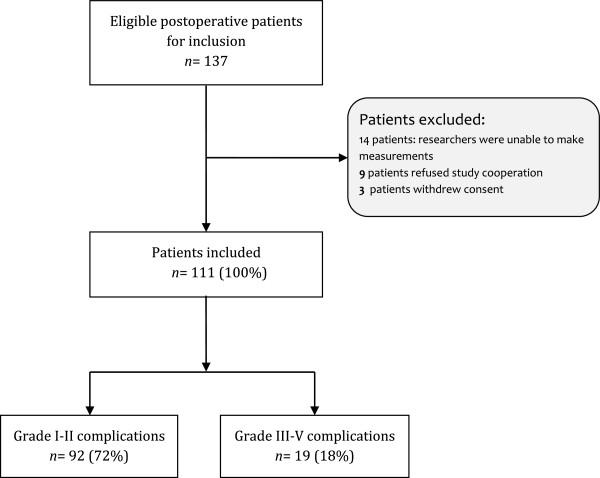
Flowchart of patient inclusion in the study.

From among the 137 patients who were eligible for inclusion during the study period, 111 patients were ultimately included (Figure 1). There were no other discontinuations or patients lost to follow-up. Table 1 summarizes the demographic, biochemical and surgical characteristics of all patients. There was no significant difference between groups.

**Table 1:** **Patients’ characteristics**^**a**^

**Characteristics**	**All patients (*N *** **= 111)**	**Nonsevere complications (*n *** **= 92)**	**Severe complications (*n *** **= 19)**
Age (yr), *median* (*IQR*)	60 (54 to 69)	60 (52 to 69)	61 (55 to 72)
Sex (males/females), *n*	76/35	62/30	14/5
Body mass index (kg/m^2^)	26.4 (0.7)	26.6 (0.8)	25.0 (1.3)
APACHE II score	13.8 (0.4)	13 (0)	15 (1)
Total SOFA score, D0	4.8 (0.3)	5 (0)	6 (1)
P-POSSUM score	31.6 (0.6)	31.0 (0.7)	33.6 (1.7)
Blood loss (ml)	1,068 (149)	971 (174)	1,493 (234)
Surgery time (min)	324 (12)	322 (13)	337 (32)
*ASA score*			
Grade I	11	11	0
Grade II	59	48	11
Grade III	40	32	8
Grade IV	1	1	0
*Type of surgery, n*			
Transthoracic oesophagectomy	22	16	6
Transhiatal oesophagectomy	13	11	2
Pancreaticoduodenectomy	23	18	5
Kidney transplantation	24	23	1
Liver transplantation	7	4	3
Hemihepatectomy	7	6	1
Partial gastrectomy	4	4	0
Hepaticojejunostomy	2	2	0
Gastroenterostomy	2	2	0
Pancreaticojejunostomy	1	1	0
Aortic bypass	1	1	0
Colon interposition	1	0	1
Colorectal surgery	1	1	0
Distal pancreaticotectomy	2	2	0
Subtotal colectomy	1	1	0

^a^APACHE II, Acute Physiology and Chronic Health Evaluation II; ASA, American Society of Anesthesiologists; P-POSSUM, Portsmouth Physiological and Operative Severity Score for the enUmeration of Mortality and Morbidity; SOFA, Sequential Organ Failure Assessment. Data are presented as the number of patients, *n* (*%*), or as the mean (SE), unless otherwise specified. Nonsevere complications category comprises no complications to grade II complications. Severe complications category comprises grades III to V complications.

### Postoperative outcomes

Of the 111 patients included, 19 patients (17%) developed severe complications (grade III, IV or V) (Table 2 and Figure 1). The mean occurrence of severe postoperative complications was 5.2 (±0.7) days. Importantly, 87% of the patients developed complications after the last peripheral perfusion measurement was obtained. The overall postoperative mortality rate was 3.6%, and the overall morbidity rate was 38.7%. Of the nonsurvivors, one patient died due to liver failure and three patients died due to infectious complications. Operative blood loss tended to be greater in patients who developed severe complications compared to patients who developed nonsevere complications, although this difference was not significant (*P* = 0.245). Fluid balance at D0 did not differ between groups (724 ± 83 ml vs. 829 ± 170 ml). ICU and hospital lengths of stay were significantly longer in patients who developed severe complications.

**Table 2:** Postoperative complications

	**All patients (*N *** **= 111)**	**Nonsevere complications (*n *** **= 92)**	**Severe complications (*n *** **= 19)**	***P*-values**
ICU stay (days), *median (IQR)*	2 (2 to 4)	2 (0 to 3)	6 (3 to 9)	<0.001
Hospital stay (days), *median (IQR)*	13 (11 to 20)	12 (10 to 17)	27 (17 to 36)	<0.001
Highest complication grade				
No complications	68	68	–	–
Grade I	11	11	–	–
Grade II	13	13	–	–
Grade IIIa	3	–	3	–
Grade IIIb	4	–	4	–
Grade IVa	6	–	6	–
Grade IVb	2	–	2	–
Grade V (death)	4	–	4	–

According to the Clavien-Dindo classification scheme, the most severe complication is scored when those of lesser severity are clearly a step in the process leading to the serious event. Data are presented as the number of patients, unless otherwise specified. *P*-values represent differences between groups, and the statistical significance threshold was set at *P* < 0.017. Dash (–) indicates not applicable. Nonsevere complications category comprises no complications- to grade II complications. Severe complications category comprises grades III to V complications.

### Systemic and peripheral perfusion parameters over time and relationship to outcomes

Haemodynamic and biological data are presented in Table 3. On the different postoperative days, there was no difference in cardiac index (*n* = 39), MAP or central venous pressure between patients who developed severe complications and those who did not. Immediately after surgery (D0), however, HR was higher in patients who developed severe complications and persisted over time until D3. In patients who received vasopressor therapy postoperatively, the dose of vasopressor did not differ between groups in linear mixed-model analysis. Overall fluid balance during the study period did not differ between groups (2,462 ± 155 ml vs. 2,879 ± 334 ml).

**Table 3:** **Systemic haemodynamic and biological data**^**a**^

	**Baseline**		**D0**		**D1**		**D2**		**D3**	
**Systemic haemodynamics**	**Nonsevere complications**	**Severe complications**	**Nonsevere complications**	**Severe complications**	**Nonsevere complications**	**Severe complications**	**Nonsevere complications**	**Severe complications**	**Nonsevere complications**	**Severe complications**
Heart rate (beats/min)	74 (1)	76 (4)	77 (1)	87 (5)^b^	80 (2)	91 (5)^b^	83 (1)	91 (4)^b^	81 (2)	92 (3)^b^
Mean arterial pressure (mmHg)	96 (2)	95 (4)	89 (2)	85 (3)	90 (2)	87 (5)	93 (2)	93 (4)	96 (1)	91 (4)
pH	7.36 (0.02)	7.41 (0.02)	7.36 (0.01)	7.37 (0.01)	7.39 (0.01)	7.39 (0.02)	7.40 (0.02)	7.43 (0.01)	7.40 (0.02)	7.43 (0.01)
PaO_2_ (kPa)	10.5 (5.8)	11.1 (0.05)	17.6 (0.9)	15.8 (1.3)	12.7 (0.5)	11.7 (0.7)	11.3 (0.5)	13.6 (2.3)	10.2 (0.6)	11.4 (1.1)
Base excess	-4.0 (2.08)	-3.0 (3.0)	-2.92 (0.29)	-2.69 (1.08)	-2.20 (0.34)	-2.0 (1.06)	-0.59 (0.47)	-0.36 (1.59)	-0.22 (0.85)	0.5 (1.29)
Lactate (mmol/L)	1.3 (0.2)	1.6 (0.01)	1.3 (0.1)	1.6 (0.3)	1.2 (0.1)	1.5 (0.2)	1.1 (0.1)	1.1 (0.1)	1.1 (0.1)	1.3 (0.2)
Haemoglobin (g/dl)	7.7 (0.2)	8.0 (0.3)	7.1 (0.1)	6.9 (0.3)	6.8 (0.1)	6.5 (0.3)	6.5 (0.1)	5.9 (0.3)^b^	6.4 (0.1)	5.8 (0.3)
Creatinine (μmol/L)	81 (7)	85 (12)	84 (6)	94 (13)^b^	76 (5)	89 (10)^b^	69 (5)	86 (9)	68 (6)	82 (10)
C-reactive protein (mg/L)	8 (2)	21 (18)	35 (9)	96 (33)^b^	86 (10)	121 (26)^b^	135 (15)	156 (24)	120 (15)	153 (29)
White blood cells (10^9^/L)	6.7 (0.3)	9.7 (2.5)	8.7 (0.4)	10.8 (1.4)	11.6 (0.5)	11.5 (0.7)	11.3 (0.4)	11.3 (0.4)	10.2 (0.5)	9.7 (0.5)
Central temperature (°C)	36.7 (0.1)	36.7 (0.1)	36.7 (0.1)	36.7 (0.1)	37.1 (0.1)	37.3 (0.1)	37.0 (0.1)	37.2 (0.1)	37.0 (0.1)	37.2 (0.1)
Vasopressor use, *n* (%)	–	–	36 (39)	11 (58)	25 (27)	10 (52)	9 (10)	7 (37)	3 (3)	3 (16)
Vasopressor dose (μg/kg/min)	–	–	0.17 (0.12)	0.07 (0.03)^b^	0.05 (0.33)	0.04 (0.02)	0.01 (0.05)	0.01 (0.07)	0	0.18 (0.01)

^a^Baseline, Prior to surgery; D0, Directly after surgery; D1 First postoperative day; D2, Second postoperative day; D3, Third postoperative day; PaO_2_, Arterial blood gas oxygen tension. Dash (–) indicates not applicable. Nonsevere complications category comprises no complications- to grade II complications. Severe complications category comprises grades III to V complications. Data are presented as mean (SE). ^b^*P* < 0.05 indicates statistical significance between groups (nonsevere complications vs. severe complications) at the specific time point. Differences between groups on the different postoperative days were assessed by linear mixed-model analysis.

Table 4 shows the time course for the different peripheral perfusion parameters. Before surgery there were no differences between groups in these parameters. However, at D0, CRT was significantly longer in patients who subsequently developed severe complications (*P* = 0.005). This difference persisted over time until D3. Similarly, there was a downward trend for PPI (increasingly altered), which reached a significant difference between groups on D2. Concurrently on D3, there was a significant difference between groups in T_skin-diff_. Importantly, mean CRT exceeded the clinical cutoff for delayed refill time (>5 seconds) at D0, whereas mean PPI and mean T_skin-diff_ values did not exceed the cutoff for abnormality.

**Table 4:** **Peripheral perfusion parameters**^**a**^

	**Baseline**		**D0**		**D1**		**D2**		**D3**	
**Measurement parameters**	**Nonsevere complications**	**Severe complications**	**Nonsevere complications**	**Severe complications**	**Nonsevere complications**	**Severe complications**	**Nonsevere complications**	**Severe complications**	**Nonsevere complications**	**Severe complications**
CRT (s)	2.4 (0.1)	2.8 (0.2)	3.6 (0.2)	5.2 (0.5)^b^	2.9 (0.2)	6.2 (0.7)^b^	2.7 (0.1)	5.9 (0.6)^b^	2.8 (0.2)	5.7 (0.7)^b^
PPI (a.u.)	3.9 (0.3)	2.9 (0.3)	3.5 (0.4)	2.7 (0.8)	4.9 (0.0)	2.4 (0.7)	3.9 (0.3)	1.7 (0.4)^b^	4.4 (0.3)	2.2 (0.5)^b^
T_skin-diff_ (°C)	2.1 (0.2)	2.5 (0.5)	2.7 (0.2)	3.1 (0.4)	2.3 (0.2)	3.3 (0.5)	2.2 (0.2)	3.9 (0.6)^b^	2.2 (0.2)	3.6 (0.7)^b^

^a^a.u., Arbitrary units; Baseline, Prior to surgery; CRT, Capillary refill time; D0, Directly after surgery; D1 First postoperative day; D2, Second postoperative day; D3, Third postoperative day; PPI, Peripheral perfusion index; T_skin-diff_, Forearm-to-fingertip skin temperature gradient. Nonsevere complications category comprises no complications to grade II complications. Severe complications category comprises grades III to V complications. Data are presented as mean ± SE for the total patient sample (*N* = 111). ^b^*P* < 0.017 indicates significant difference between groups at specific time point by linear mixed-model analysis.

**Figure 2:**
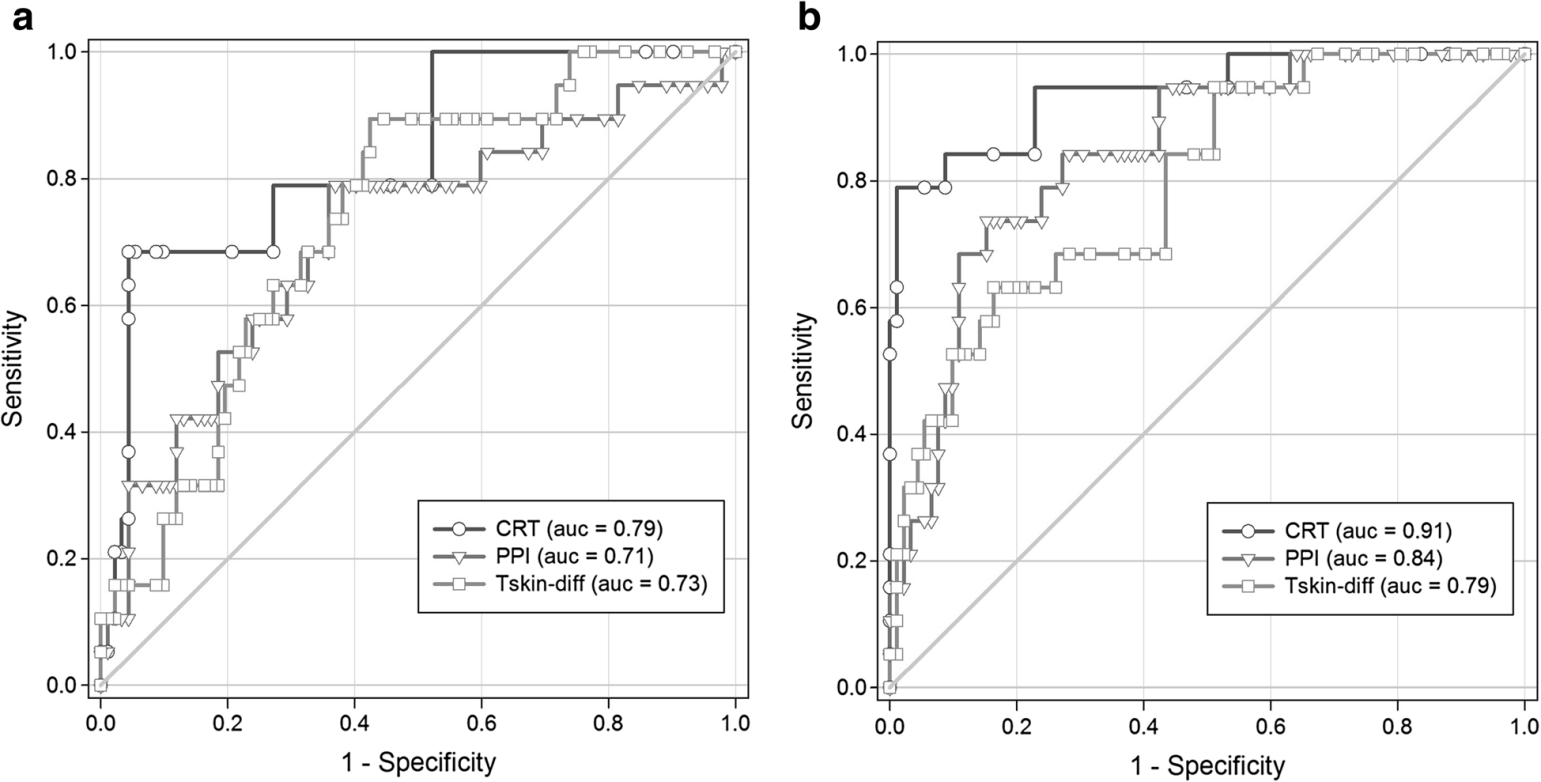
**Receiver operating characteristic curves of peripheral perfusion parameters for prediction of severe complications.** Receiver operating characteristic curves for capillary refill time (CRT), peripheral perfusion index (PPI) and forearm-to-fingertip skin temperature gradient (T_skin-diff_) to predict severe complications on the first postoperative day (D1) **(a)** and the second postoperative day (D2) **(b)**. Full details, including sensitivity and specificity values of the curves, are given in Table 5.

Peripheral perfusion parameters were treated as continuous variables and analysed accordingly over time. Amongst these different parameters, the diagnostic accuracy of predicting severe postoperative complications on D1 was highest using CRT (ROC area under the curve (AUC) = 0.79 (95% CI = 0.66 to 0.90); *P* < 0.001) (Figure 2A). This value increased marginally and had the largest AUC on D2 (0.91 (0.83 to 0.92); *P* < 0.001) (Figure 2B). Moreover, CRT was a significantly better predictor than PPI (*P* = 0.001) and T_skin-diff_ (*P* < 0.001) at D1.

For discriminatory analyses of the individual assessment of peripheral perfusion, we used predefined cutoff values for abnormal peripheral perfusion on each independent parameter. Table 5 shows the diagnostic accuracy of the different peripheral perfusion parameters for the early identification of patients with severe complications. For testing on D2, the sensitivity (0.79, 95% CI = 0.54 to 0.94) for CRT was very good, with a specificity of 0.93 (95% CI = 0.86 to 0.97). Similarly, PPI and T_skin-diff_ had the highest sensitivity (0.74 (95% CI = 0.49 to 0.91) and 0.84 (95% CI = 0.60 to 0.97), respectively) on D2 compared to the other postoperative days. Subsequently, the positive likelihood ratio obtained on D1 and D2 indicated that the accuracy of peripheral perfusion parameters improved significantly, respectively 1 and 2 days, after surgery with good predictive value. Abnormalities in peripheral perfusion parameters were already predictive on D1, and over time these abnormalities were present approximately two to twelve times more often in patients with severe complications.

**Table 5:** **Predictive value after surgery for the different peripheral perfusion parameters, when altered, as predictors of severe complications**^**a**^

**Measurement parameters**		**AUC (95% CI)**	***P*-values**	**Sensitivity (95% CI)**	**Specificity (95% CI)**	**Positive likelihood ratio**	**Negative likelihood ratio**
**CRT**	*D0*	0.76 (0.65 to 0.80)	0.31	0.63 (0.38 to 0.83)	0.79 (70 to 87)	3.06	0.46
	*D1*	0.79 (0.66 to 0.90)	<0.001	0.68 (0.43 to 0.87)	0.91 (0.83 to 0.96)	7.42	0.35
	*D2*	0.91 (0.83 to 0.92)	<0.001	0.79 (0.54 to 0.94)	0.93 (0.86 to 0.97)	11.71	0.23
	*D3*	0.88 (0.78 to 0.93)	<0.001	0.71 (0.46 to 0.89)	0.93 (0.85 to 0.98)	10.15	0.34
**PPI**	*D0*	0.59 (0.45 to 0.70)	0.19	0.42 (0.20 to 0.67)	0.64 (0.53 to 0.74)	1.17	0.90
	*D1*	0.71 (0.57 to 0.82)	0.43	0.58 (0.34 to 0.80)	0.76 (0.66 to 0.84)	2.42	0.55
	*D2*	0.84 (0.75 to 0.91)	<0.001	0.74 (0.49 to 0.91)	0.84 (0.75 to 0.91)	4.52	0.31
	*D3*	0.81 (0.70 to 0.91)	<0.001	0.53 (0.29 to 0.76)	0.90 (0.82 to 0.95)	5.21	0.53
**T**_**skin-diff**_	*D0*	0.59 (0.44 to 0.70)	0.23	0.73 (0.49 to 0.91)	0.50 (0.40 to 0.61)	1.54	0.50
	*D1*	0.73 (0.62 to 0.83)	0.14	0.82 (0.57 to 0.95)	0.58 (0.47 to 0.68)	1.91	0.36
	*D2*	0.79 (0.68 to 0.81)	<0.001	0.84 (0.60 to 0.97)	0.52 (42 to 63)	1.76	0.30
	*D3*	0.72 (0.60 to 0.80)	0.31	0.68 (0.43 to 0.87)	0.58 (0.48 to 0.69)	1.64	0.52

^a^AUC, Area under the receiver operating characteristic curve; CI, Confidence interval; D0, Directly after surgery; D1, First postoperative day; D2, Second postoperative day; D3, Third postoperative day; PPI, Peripheral perfusion index; T_skin-diff_, Forearm-to-fingertip skin temperature gradient. P < 0.017 is considered significant.

Interrater reliability of CRT between observers at the different postoperative days demonstrated a good overall agreement. Cohen’s κ analyses demonstrated κ-values of 0.91 (95% CI = 0.80 to 0.97) on D0, 0.81 (95% CI = 0.65 to 0.93) on D1, 0.74 (95% CI = 0.52 to 0.89) on D2 and (95% CI = 0.70 to 0.98) on D3. Importantly, the interrater reliability of CRT at D2, which had the best predictive value for severe complications (see Table 5), showed good agreement between the different observers.

### Accuracy of peripheral perfusion for predicting postoperative complications

We performed a generalized linear mixed-model analysis to further explore the association of abnormal peripheral perfusion with outcomes. After adjusting for haemodynamic variables (MAP and HR), vasopressor therapy, CRP and haemoglobin, the condition of abnormal peripheral perfusion was found to have a major predictive effect. The predictive value for severe complications was calculated for each postoperative day separately, and the results are presented in Table 6. Postoperatively, abnormal peripheral perfusion was predictive for development of severe complications. Notably, abnormal peripheral perfusion on D2 had the best predictive value; patients with abnormal peripheral perfusion were almost nine times more likely to develop postoperative complications (OR = 8.40 (95% CI = 2.72 to 25.87); *P* < 0.001). More importantly, this effect persisted over time. When we scored the occurrence of 30-day postoperative complications, abnormal peripheral perfusion was still predictive at the different subsequent time points: D1 (OR = 2.87 (95% CI = 1.23 to 6.71); *P* = 0.015), D2 (OR = 3.46 (95% CI = 1.46 to 8.23); *P* = 0.005) and D3 (OR = 3.34 (95% CI = 1.40 to 7.96); *P* = 0.007).

**Table 6:** **Predictive value of abnormal peripheral perfusion for severe complications**^**a**^

**Abnormal peripheral perfusion measurement**	**Odds ratio**	**(95% CI)**	***P-*** **value**
D0	4.49	1.38 to 14.56	0.013
D1	3.70	1.29 to 10.63	0.015
D2	8.40	2.72 to 25.87	<0.001
D3	6.43	2.11 to 19.64	0.001

^a^CI, Confidence interval; D0, Directly after surgery; D1, First postoperative day; D2, Second postoperative day; D3, Third postoperative day. Abnormal peripheral perfusion is defined as at least one abnormal peripheral perfusion variable *capillary refill time >4.5 seconds,* peripheral perfusion index *<1.4 or a* forearm-to-fingertip skin temperature gradient *>2°C*. We used a generalized mixed-model analysis to calculate the predictive value of abnormal peripheral perfusion for severe complications at each postoperative day. Mean arterial pressure, heart rate, vasopressor therapy, C-reactive protein and haemoglobin were used to adjust the predictors in the multivariate analysis. P < 0.017 is considered significant.

The following commonly used risk stratification scores had no predictive value for the occurrence of complications in our population: ASA score (OR = 2.62 (95% CI = 0.71 to 9.56); *P* = 0.14), P-POSSUM score (OR = 1.04 (95% CI = 0.92 to 1.18); *P* = 0.52), APACHE II score (OR = 1.11 (95% CI = 0.94 to 1.32); *P* = 0.24) and SOFA score on D0 (OR = 1.08 (95% CI = 0.81 to 1.43); *P* = 0.62).

## Discussion

The principal findings in the present study illustrate that, following elective major abdominal surgery, peripheral perfusion alterations were more marked in patients who were more likely to develop severe complications. Peripheral perfusion alterations were already present in these patients immediately after surgery and became even more predictive on D1 and D2. Further research is required to confirm these findings and to investigate whether targeted treatment based on peripheral perfusion assessment could improve outcomes.

Surprisingly, the diagnostic accuracy of peripheral perfusion assessment was highest using CRT, as compared to PPI and T_skin-diff_. This finding has major clinical implications. First, this parameter provided high levels of accuracy and discrimination, as soon as D2, in the prediction of patients more likely to develop severe complications. Second, the subjective inspection and palpation of peripheral perfusion is safe, noninvasive, cheap and easy to perform at the bedside and enables physicians to identify early on those patients more likely to deviate from the normal postoperative course and have severe complications, even before continuing to invasive procedures. Moreover, despite the current questions raised about the clinical utility of CRT, we found that it had very good interrater reliability between observers. The results of our study extend the findings of a recent single-centre study [15] which showed that subjective assessment of peripheral perfusion with CRT could identify early on those critically ill patients with more severe organ dysfunction.

Since the introduction of the Clavien-Dindo classification system in 2004 [23], an increasing consensus has been formed on how to define and grade adverse postoperative events and evaluate surgical procedures [22]. Early identification of patients who subsequently develop life-threatening complications or who are at risk for long-term disability due to postoperative complications enables timely recognition of patients who may benefit from early intensive management. Several prediction models, such as the Colorectal Physiological and Operative Severity Score for the Enumeration of Mortality and Morbidity [28], the Rotterdam score [29] and a new score described by Braga *et al*. [6], have been proposed to predict standardized complications based on several physiological and operative measurements. These prediction models are usually organ- and surgery-specific, however, and tend to overestimate poor outcomes in a surgical case mix population, and are even outranked by a surgeon’s intuition [30]. Therefore, an individual assessment, such as the assessment of peripheral perfusion, to predict postoperative patients at risk for severe adverse postoperative events could be very useful in comparing the postoperative performance of patients in a heterogeneous population. Maybe more importantly, we observed peripheral perfusion alterations before the clinical occurrence of postoperative complications, with a mean occurrence on the fifth postoperative day. Because of the preliminary nature of our findings, comparison with other measures of individual surgical risk assessment, such as exercise testing or plasma biomarkers, could confirm our observations.

From an etiological perspective, compromised peripheral circulation in the postoperative course may resemble the early, initial period of that in both septic and nonseptic shock. In the latter case, increased sympathetic activity, as a response to circulatory shock, leads to increased vasomotor tone and is usually induced by the baroreceptor reflex. During this period, in which compensatory mechanisms predominate, the neurohumoral response-induced vasoconstriction preserves the perfusion of the heart and brain at the expense of perfusion of the skin, muscle and gastrointestinal vascular beds [27,31]. On the other hand, this increased adrenergic response could also well be the result of inflammation-induced vasoconstriction due to intraoperative stress and surgical trauma, independent of systemic haemodynamics [32,33]. For instance, Boerma *et al*. showed that severe inflammation affects intestinal and sublingual tissue perfusion [34]. Furthermore, using an experimental model of abdominal surgery, Hiltebrand *et al*. found that treatment of hypotension with norepinephrine had no adverse effects on microcirculatory perfusion or tissue oxygen tension in the intestinal tract, proving that administering low to moderate doses of norepinephrine to increase perioperative blood pressure does not adversely affect peripheral tissue perfusion [35].

Others have confirmed that this postsurgical systemic inflammatory response syndrome (SIRS)–induced vasoconstriction could be seen as a surrogate marker for impaired tissue healing, increased metabolic demands and organ hypoperfusion [36,37]. In our patient population, the observed delayed CRT and increased T_skin-diff_ in patients who developed severe complications may similarly reflect the release of inflammatory mediators, leading to SIRS [38], loss of autoregulation, increased endothelial damage and tissue perfusion alterations.

Although the presence of abnormal peripheral perfusion identifies patients at increased risk for unfavourable outcomes, no study has been conducted to date, to the best of our knowledge, that has been focused on resuscitation based on these parameters. Current perioperative ‘goal-directed therapy’ studies have been focused mainly on systemic haemodynamic parameters [11,39] and may contribute to improvements in survival after major surgery by using perioperative plasma volume expansion [12,39] or a more restricted fluid approach [40]. Moreover, there is to date no uniform goal-directed strategy aimed at resuscitation of regional peripheral blood flow. Some researchers have shown positive effects of regional perfusion-based resuscitation [41,42], whereas others have reported contrasting findings [43]. Our findings in the present study have important clinical implications, however, as our results demonstrate the importance of adequate tissue perfusion and thus may provide a foundation for developing a tissue perfusion-based approach for use even before critical illness [44,45]. The role of individual peripheral perfusion assessment as a potential additional tissue perfusion endpoint must be investigated further to address whether aiming at normalization has an impact on outcome. Given the results we report here, one can imagine that serial peripheral perfusion measurements in a postoperative population could provide an additional window to monitoring and treating occult hypoperfusion caused by inflammation or infection. As such, these measurements might be used for earlier treatment of surgical or infectious postoperative complications and thereby result in a reduction of organ failure and mortality. However, a large multicentre trial, preferably randomized and controlled, is needed to demonstrate the effect of interventions based on impaired peripheral perfusion parameters.

Some limitations of our study need to be acknowledged. First, because our study was an observational study, significant correlations between peripheral perfusion alterations and the occurrence of complications do not prove causality. It has been shown previously, however, that persistent vasoconstriction, independently of systemic haemodynamics, suggests ongoing sympathetic activity associated with organ dysfunction after out-of-hospital cardiac arrest [16] and during septic shock [17,46]. As such, there is now evidence that an elevated SIRS response before and directly after major surgery (within 24 hours) is associated with increased morbidity [9,47] and mortality [48] and is an independent predictor of survival.

Second, in this study we chose to correlate peripheral hypoperfusion to clinical outcomes according to the Clavien-Dindo grading system [23]. Although this grading system is relatively new, it is not unfamiliar amongst surgeons and it is considered more reliable by observers [22]. This classification system is simple and reproducible, correlates with treatment cost and length of stay and can be used to semiquantitatively score postoperative complications in severity in varying fields of surgery. Although 30-day follow-up is often used, we focused on a 10-day time window. We believe that the scoring of complications within 10 days after surgery better permits distinctions between procedure-related complications and those related to disease progression, especially when related to perioperative perfusion abnormalities. Nevertheless, we found that the predictive effect of impaired peripheral perfusion persisted over 30 days as well.

Third, paired measurements of global blood flow (cardiac output) were not done in all postoperative patients. Our main focus in this study was to assess the relationship between abnormal peripheral perfusion and the occurrence of severe complications. It remains to be elucidated to what extent peripheral perfusion alterations reflect systemic haemodynamics, as others have reported that peripheral circulation behaves independently of systemic haemodynamic resuscitation, even despite early goal-directed therapy [18,46]. Despite this knowledge, we observed a significant difference in HR between groups at the different postoperative time points. Although HR is indeed an important prognostic parameter, our objective in this study was to examine whether peripheral perfusion assessment could improve risk stratification, and we therefore corrected for HR in the multivariate analysis.

We did not include other methods of peripheral blood flow monitoring, such as cutaneous laser Doppler flowmetry. We previously showed that CRT, PPI and T_skin-diff_ are well-validated methods of estimating cutaneous blood flow [15] and provide a ready-to-use, simple, cheap instrument at the bedside. Moreover, real-time evaluation of the (subjective) assessment of peripheral perfusion is easily obtainable by using noninvasive monitoring techniques, and it can be rapidly applied throughout the hospital under different circumstances and by different health-care workers.

## Conclusions

By simple clinical assessment of peripheral perfusion immediately after surgery, clinicians are able to discriminate patients at high risk for developing severe complications. These findings suggest that assessment of peripheral perfusion can be used for early identification of postoperative patients in need of additional therapy and open a perspective on new (tissue perfusion) goal-directed therapies. Further research designed to confirm these findings, and to explore them in more detail, is needed. Such research needs to be conducted in a powered, randomized, controlled fashion to assess the effects on outcome of a peripheral perfusion targeted resuscitation following major abdominal surgery.

## Key messages

• Peripheral perfusion assessment improves risk stratification independently of systemic haemodynamics.

• Simple clinical assessment of peripheral perfusion can be used immediately after surgery to discriminate patients at high risk for developing severe complications.

• Monitoring of peripheral tissue perfusion might be a valuable adjunct for use in identifying patients who are eligible for additional therapy, with the aim of recruitment of the peripheral perfusion.

• Further prospective randomized controlled evaluation is needed to determine the role of peripheral perfusion assessment in the early individual postoperative goal-directed therapy and its effect on outcome.

## Abbreviations

APACHE: Acute Physiology and Chronic Health Evaluation; ASA: American Society of Anesthesiologists; AUC: Area under the receiver operating characteristic curve; BL: 1 day before surgery; CRT: Capillary refill time; D0: Directly after surgery; D1: First postoperative day; D2: Second postoperative day; D3: Third postoperative day; HR: Heart rate; MAP: Mean arterial pressure; PPI: Peripheral perfusion index; P-POSSUM: Portsmouth Physiological and Operative Severity Score for the enUmeration of Mortality and Morbidity; ROC: Receiver operating characteristic; SOFA: Sequential Organ Failure Assessment; T_skin-diff_: Forearm-to-fingertip skin temperature gradient.

## Competing interests

The authors declare that they have no competing interests.

## Authors’ contributions

MvG designed the study, analysed and interpreted the data and drafted the manuscript. JP conducted the study. JdJ participated in data interpretation and helped to draft the manuscript. RvdV analysed and reviewed the data. AL assisted in study design, data analysis and data interpretation. JB assisted with study design. JvB conceived the study, participated in its design and coordination and reviewed the manuscript. All authors read and approved the final manuscript.
